# Antecedent configuration pathways for manufacturing-enterprise digital servitization: Based on a technology-organization-environment theoretical framework

**DOI:** 10.1371/journal.pone.0301789

**Published:** 2024-05-22

**Authors:** Xu Zhang, Huijuan Zhao, Weijie Zhou

**Affiliations:** 1 College of Economics and Management, Qingdao University of Science and Technology, Qingdao, China; 2 College of Economics and Management, Shandong University of Science and Technology, Qingdao, Shandong, China; 3 College of Finance and Economics, Shandong University of Science and Technology, Taian, Shandong, China; Harbin Institute of Technology, CHINA

## Abstract

The expeditious advancement and elevation of the manufacturing industry’s transformation and upgrading represent pivotal strides for China in its ascent toward the upper echelons of the global manufacturing value chain. Currently, China’s manufacturing-industry transformation faces the dual-lag quandary of digitalization and servitization. The notion of digital servitization elucidates the interdependent relationship between digitalization and servitization, unveiling the mechanisms underlying the formation of digital servitization. This holds significant implications for advancing the comprehension of digitalization and servitization and, crucially, facilitates the acceleration of China’s manufacturing sector transitioning from production-centric to service-centric paradigms. Harnessing the technology-organization-environment (TOE) theoretical framework, we constructed a model elucidating the driving factors underpinning manufacturing digital servitization. By employing the fuzzy-set qualitative comparative analysis (fsQCA), we explored strategic decisions and path dependencies in the transformation of manufacturing digital servitization, offering valuable insights to foster China’s manufacturing sector in its digital-servitization journey. The following findings were obtained. (1) A singular condition was insufficient as a prerequisite for manufacturing digital servitization and necessitated the coordinated alignment of multiple variables. (2) Three pathways existed for achieving manufacturing digital servitization: TOE, organization-environment collaborative-oriented, and technology-organization collaborative-oriented. (3) The progression of manufacturing digital servitization resulted from the collective impact of numerous factors, exhibiting a characteristic of different paths leading to the same destination. Various manufacturing enterprises pursued distinct trajectories to achieve digital servitization, contingent upon their unique circumstances. These findings have the potential to provide valuable insights for effectively fostering manufacturing digital servitization.

## 1. Introduction

In the digital-economy era, the manufacturing industry is undergoing a profound metamorphosis. Propelled by emerging technologies such as big data, the Internet of Things (IoT), blockchain, and artificial intelligence (AI), the digital surge is fundamentally reshaping organizational structures [1], team collaborations, operational management, and the business mindset of manufacturing enterprises [2]. With manufacturing-enterprise business models transitioning toward a consumer-centric service-oriented paradigm [3], scholars have extensively investigated the emergence of the servitization concept. Concurrently, the continual advancement of novel digital technologies such as big data, the industrial IoT, and AI, alongside the digital platforms constructed upon them, is propelling a wave of digitization across diverse industries [4]. Certain scholars, amid their examination of the servitization process, have recognized that digitization serves as a potent catalyst for augmenting service efficiency [5]. Additionally, digitization has been acknowledged as a pivotal force propelling enterprise business models, value capture, and value creation [6].

Within the realm of digitization research, the utilization of emerging digital technologies is recognized as a potent avenue for fostering innovative services, thus catalyzing the evolution of servitization and bolstering service innovation performance [7]. The examination of servitization and digitization is transitioning from fragmentation toward integration, heralding the emergence of a novel domain: digital servitization [8]. Digital servitization, serving as a fundamental strategy propelling the enhancement of manufacturing enterprises, has garnered considerable attention [9]. In contrast to the evolution of traditional servitization, digital servitization demands heightened organizational agility and flexibility in work methodologies [10]. This transformation endeavors to elevate the production and service models of conventional manufacturing enterprises to intelligent, efficient [11], and adaptable models grounded in digital technology. Nevertheless, to attain digital-servitization transformation, enterprises require deep comprehension and effective management of the intricate interplay among diverse factors.

Confronted with the global surge of digitization, the manufacturing sector must not only promptly adjust to technological shifts but also exhibit adaptability in navigating varied market landscapes and evolving societal needs [12]. As they endeavor to capitalize on new technologies and market prospects, manufacturing enterprises must undergo a significant transformation, reshaping their operational paradigms [13]. Nonetheless, attaining the objective of digital-servitization transformation lacks readily accessible guiding frameworks. A profound comprehension of the precursor configuration pathways of digital-servitization transformation is essential for the manufacturing industry to attain sustainable competitiveness and foster innovative development.

Against this backdrop, the primary objective of this paper is to scrutinize the driving forces behind digital servitization in manufacturing enterprises. To expand the investigation into the precursor factors of digital servitization in manufacturing, we utilized the qualitative research approach of fuzzy-set qualitative comparative analysis (fsQCA) to examine the methods through which Chinese manufacturing enterprises accomplish digital service-oriented transformation. Comprehensive exploration of the precursor configuration pathways of digital-servitization transformation is indispensable for gaining a deep understanding of its implementation mechanism, thereby furnishing manufacturing enterprises with an effective strategic direction to accomplish digital-servitization transformation within the digital-economy era.

## 2. Literature review and theoretical framework

### 2.1. Digital servitization

The advent of digitalization has ushered in novel modes of interaction between manufacturing enterprises and their clientele. Industrial Internet platforms, harnessed by digital technologies, render feasible the endeavor of manufacturing enterprises to partake in the collaborative creation of value with their clientele [14]. This aids manufacturing enterprises in comprehending customer needs, crafting tailor-made products to fulfill those needs, and fostering service innovation [15]. With the transition of customer demands from mere acquisition of consumer goods to embracing intricate solutions for meeting expectations and engendering utility [16], the dynamics between customers and the commercial sphere have evolved from the traditional product-centric approach of manufacturing enterprises to a consumer-centric, service-oriented paradigm. Manufacturing enterprises actively interact with customers, capturing their existing needs and probing potential ones to amass digital resources [17]. This process establishes a dynamic foundation of resources to fortify core competitiveness, fundamentally revamping the value chain and steering the process of customer value creation.

Scholars like Lu et al. [18] elucidated that digitalization aids enterprises in acquiring and analyzing data, augmenting customer perception, and attaining personalized customization along with networked collaborative transformation. Notwithstanding the progressive universally applicable and integrative nature of digital technology, which proffers novel avenues for value creation [19], digitization is exerting a profound impact on industries, society, and the economy [20]. Nevertheless, extant research on digitization has yet to distinctly delineate how manufacturing enterprises harness emergent digital technologies like big data, the IoT, and industrial Internet platforms to refine products, iterate business models, and reshape the dynamics of value creation.

With a substantial portion of manufacturing enterprises transitioning toward a consumer-centric, service-oriented paradigm, short-term coordination challenges may stem from internal resource allocation, notably escalating operating expenditures and managerial complexities, giving rise to the phenomenon of the servitization paradox [21]. Over the long haul, excessive servitization may culminate in a dissociation between service-oriented endeavors and tangible products, precipitating augmented managerial expenses, heightened operational hazards, and a debilitation of the enterprise’s capacity for innovation [22], thus impeding the enhancement of total factor productivity.

Within the realm of research concerning the interplay between digitization and the transformation toward servitization in the manufacturing sector, certain studies have proposed that the advancement of digitization hinges upon the requisition for servitization within manufacturing. These studies have posited that digitization, intelligence, and other traits of informatization are representations of sophisticated service inputs in the realms of knowledge and technology for manufacturing enterprises [23]. Concurrently, as manufacturing servitization progresses, demand will grow for top-tier information services [24]. Moreover, alternative studies have posited that digitization assumes a pivotal facilitative function in the realm of manufacturing servitization. As such, by deploying digital technology, intelligent manufacturing techniques, and Internet-based business models, digitization within the manufacturing sector can amalgamate service-provisioning capabilities, cater to the diversity of service demands [25], and foster technological and service innovations conducive to manufacturing servitization. Nonetheless, the evolution of manufacturing digitization and servitization constitutes a relationship that transcends mere causality. Digitization evolves in response to the exigency for servitization within manufacturing [26]; concurrently, it propels the advancement of servitization in manufacturing [27]. Both processes are mutually dependent during their development, underscoring the necessity of interaction and collaboration.

Some scholars have investigated the co-evolving relationship between manufacturing digitization and servitization. Nevertheless, extant research has predominantly relied on scenario analysis, devoid of substantial empirical evidence. Historically, investigations into manufacturing digitization and servitization have operated as distinct domains until the advent of "digital servitization" in recent years, which has garnered considerable attention in the global arena of servitization research. Expanding upon the research conducted by Chen and Gao [28], this study delineated digital servitization as the utilization of digital technology by enterprises to reconfigure existing organizational structures, business models, and value-creation mechanisms via personalized communication modalities within a service-oriented framework. This definition recognizes the fundamental role of digital technology in driving the shift toward servitization while elucidating the inherent rationale of digital servitization, which entails the reconfiguration of mechanisms for aligning and linking supply with demand.

Prevailing research on manufacturing digital servitization has predominantly centered on delineating the concept [29], with particular emphasis on elucidating the strategic significance of digital servitization and its catalytic impact on organizational resilience. Additionally, it has explored the ways in which digital servitization fosters innovation in business models and subsequently influences the logic of value creation. Chen and Gao acknowledged that digital servitization is not just an amalgamation of digitization and servitization but also hinges on emerging digital technologies to reshape the transformation paradigm of supply-demand matching modes [30]. Meng et al. [31] posited that a judicious arrangement of digital servitization strategy has the potential to bolster organizational resilience in navigating uncertain environmental shifts, underscoring the pivotal role of digital-servitization strategy in propelling enterprise performance. Of note is the conclusion of Jian et al. [8] that digital servitization constitutes an efficacious approach for manufacturing enterprises to engender value, enabling them to reconfigure business models, assert diverse product-service value propositions, and augment customer retention. Nevertheless, scant scholarly attention has been devoted to probing the driving forces behind the transformation of digital servitization. Hence, we employed the fsQCA method to unravel this research inquiry, aiming to answer the following question: what are the antecedent driving factors of digital-servitization transformation in manufacturing enterprises in the era of the digital economy?

### 2.2. Technology-organization-environment framework

The technology-organization-environment (TOE) framework posits that the application of technology is influenced by multiple factors within and outside the TOE context. The TOE framework exhibits broad applicability and explanatory power in various contexts. For instance, Katebi et al. [32] utilized the TOE framework to analyze the driving factors to use prefabricated concrete in the field of construction engineering. The framework, incorporating both internal and external organizational factors, was used to effectively explain the multifaceted drivers for the intention to use prefabricated concrete. Ng et al. [33], based on the TOE framework, revealed internal and external factors affecting remote work and work-life balance in the COVID-19 pandemic. Moreover, Kouhizadeh et al. [34] investigated internal and external factors influencing blockchain adoption, employing the TOE framework to uncover technological, organizational, and environmental contextual factors as obstacles to blockchain adoption.

**Fig 1:**
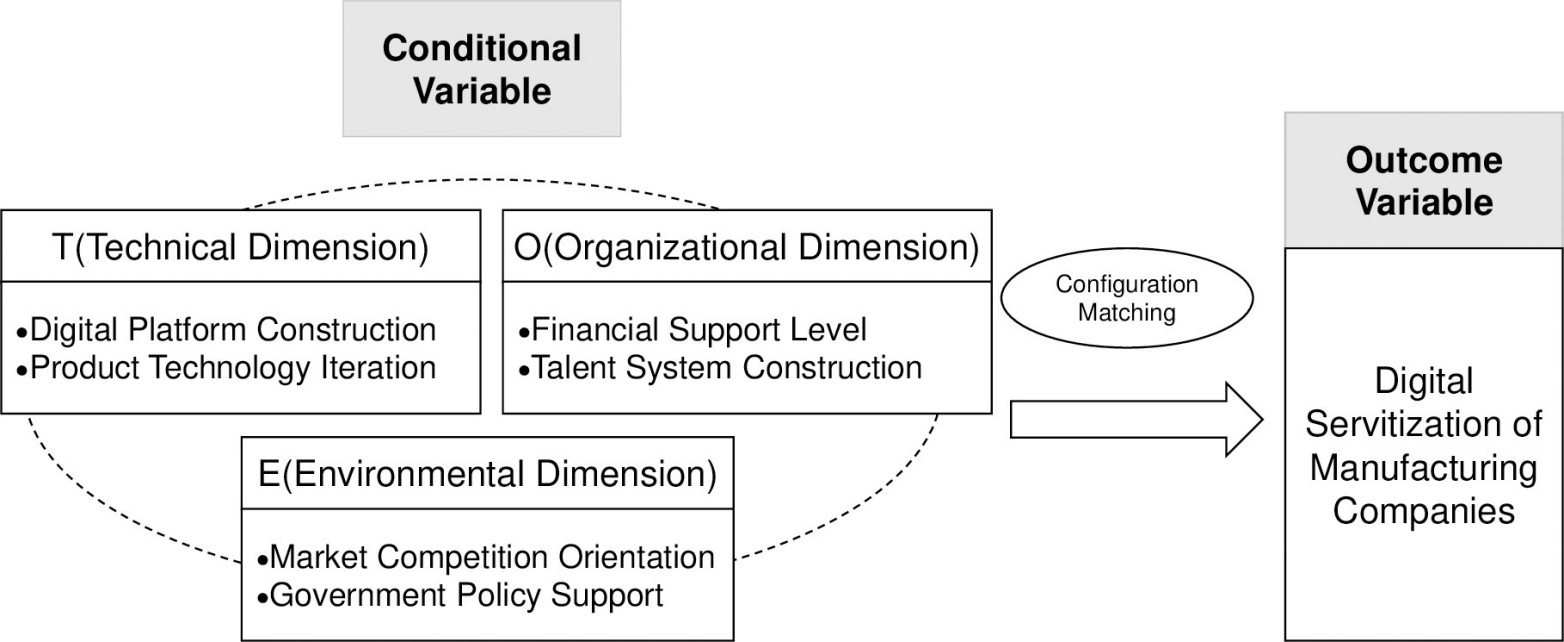
Analytical framework of digital servitization.

In this study, drawing inspiration from Baker’s research [35], we employed the TOE analysis framework, coupled with the specific context and practical scenarios of Chinese manufacturing enterprises’ transformation. We identified TOE contexts as driving factors for the digital-servitization transformation activities of manufacturing enterprises. Building upon this, we constructed a model of the antecedent driving configuration pathways for the digital-servitization transformation of manufacturing enterprises, as illustrated in **Fig 1**.

1. Technological Context: Technological progress forms a crucial foundation for the digital-servitization transformation of manufacturing enterprises. This is primarily manifested in two aspects: the establishment of digital platforms for manufacturing enterprises [36] and the iterative advancement of product technology [37]. It serves as a key pathway for achieving the transformation of manufacturing enterprises. In the digital era, manufacturing enterprises actively embrace emerging digital technologies such as big data, the industrial Internet, and AI to build digital platforms. This enhances interaction with customers, generates a wealth of consumer data, accurately identifies customer needs, and explores potential customer demands, in addition to innovating and transforming the traditional product-centric logic into a customer-centric service-oriented logic. Product-technology iteration refers to manufacturing enterprises leveraging industrial Internet platforms to enhance user catalysis and market-development trends. Thus, these enterprises absorb emerging technologies into product development, continuously iterate and improve, and better meet customer demands [38].
2. Organizational Context: Organizational management is a crucial internal transformation for the digital-servitization transformation of manufacturing enterprises. This is primarily reflected in two aspects: the level of financial support and the construction of a talent system. These aspects constitute the internal driving forces for steering the transformation of manufacturing enterprises. Confronted with intricate environmental shifts, manufacturing enterprises elevate the level of financial backing, actively pursue internal organizational alterations, fortify strategic transformational leadership, perpetually innovate, and strive to bolster the inherent competitiveness of the enterprise to cope with the uncertainty of the current era of volatility, uncertainty, complexity, and ambiguity. The digital-servitization transformation of manufacturing enterprises is a complex endeavor, requiring a substantial pool of digital, research and development, and management talents as indispensable human resources for the enterprise. Redistributing the roles of diverse talent and establishing a comprehensive and systematic talent-management system is imperative.
3. Environmental Context: Environmental support presents a pivotal opportunity for the digital-servitization evolution of manufacturing enterprises. It is primarily reflected in two aspects: market-competition orientation and government policy support. These are indispensable conditions for enterprises to achieve “overtaking on the curve,” which refers to keeping up with and eventually overtaking foreign competition. Moreover, market-competition orientation reflects the development basis of competition between enterprises and encourages enterprises to fully utilize digital technology to grasp the direction of development, break through bottlenecks, produce products that better cater to customer needs, and enhance market competitiveness [39]. Additionally, amid government policy support, comprehensive policy guidance necessitates manufacturing enterprises to foster positive information interaction with customers [40]. This involves in-depth analysis of constantly changing consumer demands, optimizing iterative products, and producing new products that satisfy customer needs, thus enhancing customer stickiness [41].

## 3. Materials and methods

### 3.1. FSQCA method

We employed the fsQCA method, proposed by Ragin et al. [42], to analyze the antecedent configurations of digital-servitization transformation in the manufacturing industry. The rationale for selecting this method is as follows. (1) The fsQCA method, grounded in Boolean algebra and set-theory principles, constructs configurations by amalgamating diverse conditional variables, addressing research inquiries from a configurational standpoint. It scrutinizes the causal associations among various combinations of conditional variables and the outcome variable, analyzing predominant pathways that substantially influence the outcome variable and formulating an explanatory model [43]. (2) In contrast to regression-analysis methods, which are limited to elucidating symmetric issues, fsQCA represents an asymmetric data-analysis approach. In economic and social realms, numerous asymmetric matters arise that are beyond the analytical scope of linear-regression methods. The fsQCA method contends that sets of conditional variables, rather than individual factors, exert influence on the outcome variable. (3) fsQCA is adept at examining random yet intricate small-sample data (applicable to samples ranging from 10 to 100) and enables simultaneous analysis of the collective impacts of multiple factors while accommodating the intricacies of causal relationships.

### 3.2. Indicator selection and data sources

The study selected manufacturing companies listed on the Shanghai and Shenzhen stock exchanges (A-shares) and the New Third Board as research samples. The sample-selection criteria, guided by Buer et al. [44], included manual identification based on the main business, industry analysis, and exclusion of ST, PT, S*ST, and companies with missing data. Following screening, a total of 28 sample companies meeting the criteria were acquired, including 16 A-share companies and 12 New Third Board companies. The sample data for these companies are from the year 2022, and the research data primarily come from the CSMAR database, the National SME share-transfer system, and various annual corporate reports.

1. Digital Servitization (DS): Three items measure digital servitization: “identity transformation,” indicating fundamental changes in the company’s digital identity and self-awareness of its core business; “dematerialization,” indicating the increasing role of data and information in manufacturing enterprises; and “collaboration,” indicating extensive collaboration between the enterprise and customers and partners. We measured digital servitization by referencing the approach of Tronvoll et al. [2], modifying the process to align with the Chinese management context. Taking into account data accessibility and questionnaire efficiency, we ensured that all items adopted a 7-point Likert Scale (1 indicating strongly disagree, 7 indicating strongly agree) and were gathered via online surveys. Exploratory factor analysis and common-method-bias validation methods were applied to ensure the reliability of the questionnaire data.
2. Digital Platform Construction (DPC): The measurement entails a virtual variable indicating whether a manufacturing enterprise adopts a digital platform, following the methodology of Yang and Liu [45]. It was identified based on substantial participation in digital platforms, such as investments. The variable was assigned a value of 1 if the company engaged in digital platforms and 0 otherwise.
3. Product Technology Iteration (PTI): Research and development investment serves as a partial indicator of the extent of product technology iteration. Following Zheng et al. [46], we gauged the product-technology iteration by the ratio of research and development expenses to operating income.
4. Financial Support Level (FSL): The SA index, adopted from Hadlock and Pierce [47], measures the company’s financial support level using the formula $SA=-0.737\times Size+0.043\times {Size}^{2}-0.04\times Age$. Here, size denotes the logarithm of total assets, and age indicates the number of years since the company’s IPO. Given the negative SA value, its absolute value was considered, with a greater value indicative of reduced financing constraints and heightened financial support.
5. Talent System Construction (TSC): The percentage of employees holding a bachelor’s degree or higher serves as a metric for assessing talent-system construction. The metrics for evaluating talent-system construction were drawn from prior research, with the percentage of staff holding a bachelor’s degree or higher being indicative of the efficacy of talent-pool development and the scientific research and innovation capabilities of enterprises. Undoubtedly, personnel with a bachelor’s degree or above represent the research and innovation capabilities of enterprises to a certain extent.
6. Market-Competition Orientation (MCO): The measurement of market competition entailed the calculation of the Herfindahl-Hirschman Index (HHI) based on revenue. HHI ($HHI=\sum_{i=1}^{N}{\left(\frac{X_{i}}{\sum X_{i}}\right)}^{2}$, where X_i_ is the sales of company and i) reflects industry-competition intensity inversely. A higher value signifies greater industry competition.
7. Government Policy Support (GPS): The government serves as a crucial organizational factor in advancing digital service-oriented manufacturing enterprises. Government policy support assists in directing manufacturing enterprises to engage in digital service-oriented endeavors while also contributing to the promotion of high-quality development in the national economy through avenues like financial support, tax incentives, and policy incentives. Government policy support is evident in government subsidies, quantified by the ratio of total government subsidies to total assets at the conclusion of the period.

## 4. Results

### 4.1. Variable calibration

We chose research variables and employed three qualitative anchor points for structured calibration. Specifically, the thresholds for full membership, full non-membership, and the crossover point were set at the 95th, 5th, and 50th percentiles, respectively. The data were transformed into membership degrees ranging from 0 to 1 using fsQCA4.1 software. To resolve the exclusion of cases precisely at 0.5 membership, in accordance with the recommendation of Crilly et al. [48], we adjusted cases with a membership degree of 0.5 to 0.501 or 0.499 to preserve case integrity. The comprehensive outcomes of variable calibration are delineated in **Table 1**.

**Table 1:** Calibration of conditional variables and results.

Variables	Indicators	Description of indicators	Fully belonging points (0.95)	Fully crossover points (0.5)	Fully not belonging points (0.05)
Result variable	DS	Standardized mean of 3D items	6.772	4.675	1.497
Conditional variable	DPC	Grouping based on the digital-platform-investment situation	/	/	/
	PTI	The ratio of research and development expenses to operating income	0.125	0.052	0.002
	FSL	Measure with SA index	4.190	3.500	3.304
	TSC	Percentage of employees with a bachelor’s degree or higher	0.721	0.214	0.096
	MCO	Reciprocal of hhi (herfindahl-hirschman index)	64.215	16.959	4.566
	GPS	Ratio of government subsidies to total assets	5.889	2.433	0.096

### 4.2. Necessary-condition analysis

Before performing the configurational analysis on the truth table of influencing factors, it is imperative to assess the necessity of each antecedent condition. A consistency level exceeding 0.9 for a specific antecedent condition designates it as a requisite condition for the outcome variable. In the analysis of antecedent condition consistencies, no antecedent variable exhibited a consistency level surpassing 0.9. Consequently, this result suggests the absence of indispensable antecedent variables, and the digital servitization of manufacturing enterprises arises from the synergistic interplay of multifaceted factors encompassing technology, the organization, and the environment, as depicted in **Table 2**.

**Table 2:** Necessity analysis of individual conditions.

Condition Variables	High-level DS	
	Consistency	Coverage
High DPC	0.640223	0.880383
Non-high DPC	0.398052	0.325926
High PTI	0.581072	0.577056
Non-high PTI	0.590118	0.626755
High FSL	0.783577	0.809955
Non-high FSL	0.477383	0.486594
High TSC	0.693807	0.724564
Non-high TSC	0.520529	0.525281
High MCO	0.402227	0.399447
Non-high MCO	0.773139	0.821138
High GPS	0.590118	0.710218
Non-high GPS	0.595685	0.533001

### 4.3. Portfolio analysis of influencing factors

The quantity of cases acts as a filtering criterion for particular configurations entering Boolean minimization calculations. Given the classification of the sample size as “small”, this study established a case-frequency threshold of 1. The initial consistency threshold was determined as 0.80, with the PRI threshold set at 0.75. Consequently, PRI values in the results column below 0.75 were corrected to 0. Throughout the analysis, three types of solutions emerged: complex, intermediate, and simple solutions. Leveraging insights from existing research, we employed intermediate solutions to ascertain the quantity of configurations leading to the outcome and the associated conditions. Subsequently, the outcomes of the reduced solutions aided in identifying the core conditions relatively crucial for a specific configuration. The antecedent configurations for the digitization of manufacturing enterprises are depicted in **Table 3**. Given that the overall consistency surpassed 0.8 and that the overall coverage exceeded 0.5, we can infer a high degree of consistency and explanatory capability, rendering the analysis outcomes dependable.

**Table 3:** Analysis of antecedent histories of high-level digital servitization.

Condition Variables	high-level DS			
	Y1	Y2	Y3	Y4
DPC	●	⊗	●	●
PTI	●	⊗		•
FSL	•	●	●	●
TSC	●		•	●
MCO	⊗	•	⊗	
GPS	•	●		
Coverage	0.4639	0.3891	0.2346	0.3867
Unique Coverage	0.1809	0.1080	0.0527	0.0170
Raw Consistency	0.9796	0.9762	0.9816	0.9846
Solution coverage	0.7527			
Solution consistency	0.9541			

Note:●means that the core condition exists, •means that the edge condition exists, ⊗ means that the core condition (and the edge condition) is missing, and a space means that both are missing.

In a vertical comparison of the four configuration paths, Configuration 1 followed a TOE-oriented pathway, Configuration 2 followed an “organization-environment collaborative-oriented pathway,” and Configurations 3 and 4 were “technology-organization collaborative-oriented pathway,” with each configuration elucidating 46.39, 38.91, 23.46, and 38.67% of the sample enterprises accomplishing digitization, respectively. Moreover, variables from the technological, organizational, and environmental contexts manifested in a harmonized manner across the four configurations. This suggests that no individual variable possesses the capability to propel the digitization transformation of manufacturing enterprises alone; rather, a collaborative interplay of multiple variables is imperative to advancing the manufacturing industry’s service transformation process.

Through a horizontal comparison of the four configuration paths, it became apparent that the digitization of manufacturing enterprises was contingent upon specific pathways. Configuration 1 underscored a TOE-guided approach, while Configuration 2 centered on an “organization-environment” collaborative approach, and Configurations 3 and 4 accentuated a “technology-organization”-oriented pathway approach. Core conditions in these configurations encompassed the establishment of a digital platform and the level of financial support, underscoring their pivotal role as primary catalysts for manufacturing industry digitization transformation.

### 4.4. Robustness analysis

Within fsQCA methods, three common approaches are employed to conduct robustness tests, comprising adjustments to calibrated qualitative anchors, alterations in case frequencies, and increases in the consistency threshold [49]. Owing to the scarcity of qualitative research on digitization, this study lacked theoretical underpinnings for adjusting calibration anchors. Furthermore, considering the moderate sample size of 28, altering case frequencies could potentially introduce bias into the configuration results. Consequently, the chosen approach for robustness testing in this study involved elevating the consistency threshold [50]. While maintaining other procedures constant, we gradually increased the consistency threshold from 0.80 to 0.85 and 0.90. The results remained consistent, indicating the robustness of the configuration outcomes.

## 5. Discussion

Of the four aforementioned configurations, Configurations 3 and 4 exhibited identical core conditions, rendering them second-order equivalent configurations. To underscore the distinctions between configurations and align with the TOE framework, this study designated the three types of configurations as “TOE-oriented pathway,” “organization-environment collaborative-oriented pathway,” and “technology-organization collaborative-oriented pathway.” Subsequently, amalgamating theory with pertinent cases, we undertook an analysis of the intrinsic meanings within each category of high-level digitization configurations.

### 5.1. TOE-oriented pathway

Configuration 1 attained high-level digitization through core conditions including robust digital-platform construction, advanced product-technology iteration, strong financial support, and well-established talent systems, with substantial government policy support as an additional condition. Configuration 1 posits that despite uncertain market competition and insufficient government policy support, manufacturing enterprises can attain digitization through their own technological advancements and organizational management strategies. Specifically, digitization constitutes an unpredictable endeavor where early preparation is paramount. Amid external competitive pressures, manufacturing enterprises propel the integration of digital technology and organizational structures, transitioning from traditional organizational paradigms to more adaptable and flexible digital frameworks [51]. This enables them to gain access to a more comprehensive array of innovation insights, enhance the efficacy of innovation resource utilization, and adapt to the digital milieu.

An illustrative instance of Configuration 1 is Haier, in the niche of home-appliance manufacturing. Haier stands as a global frontrunner in furnishing solutions for enhanced living standards and digital evolution. Confronted with diminished governmental backing in Qingdao relative to prominent urban centers like Beijing, Shanghai, and Guangzhou, coupled with heightened market competitiveness and inadequate local governmental assistance, Haier embarked on investing in the COSMOPlat for the execution of digital transformation initiatives. Consequently, year-on-year growth rate of research and development investment as a proportion of revenue amounted to 20.15% from 2021 to 2022. Concurrently, the enterprise enlisted multifaceted professionals possessing adeptness in industrial domains and prowess in Internet technology. Subsequently, they constructed an information-dissemination platform tailored to actual business requisites, thereby fostering data circulation and collaborative endeavors spanning diverse departments. Furthermore, they instituted a collaborative innovation platform for research and development in manufacturing, fostering connections among suppliers, partners, and consumers. This continual deepening of digital transformation has resulted in sustained enhancements in operational efficiency and continuous refinement of user experience.

### 5.2. Organization-environment collaborative-oriented pathway

Within Configuration 2, the core conditions encompassed an elevated assurance level of funding and robust governmental policy support, while heightened market-competition orientation served as an edge condition. Configuration 2 indicates that when the market-competition environment is intense, manufacturing enterprises can achieve digitization by optimizing internal organizational changes, regardless of technological advancements. In particular, amid intensified external-market competition and heightened innovation dynamism across the industry, the exigencies of market competition necessitate manufacturing enterprises to harbor personalized production and service competencies. The amalgamation of digital-resource restructuring and the integration of internal and external resources by manufacturing entities into novel business paradigms augments the competitive and collaborative dynamics among enterprises within the platform innovation ecosystem [52]. Thus, Configuration 2 substantiates the beneficial impacts of organizational metamorphosis and environmental backing on digital service innovation within manufacturing enterprises, elucidating the synergistic interplay between organizational governance and environmental support in propelling digitization.

An Illustrative instance emblematic of Configuration 2 is Chongqing Melng Electric Co., Ltd., in the realm of electrical machinery and equipment manufacturing. Confronted with tepid domestic market demand, exemplified by a 2.2% year-over-year downturn in the retail sales of home appliances from 2018 to 2019, the enterprise embarked on internal reform initiatives and devised and executed a “smart” strategy. Vigorously assimilating technologies including industrial networks, 5G, AIOT, and edge computing into the manufacturing sphere, the enterprise attained interconnectedness, cloud-based equipment, and cloud-centric operations, thereby catalyzing the elevation of product manufacturing toward enhanced flexibility, intelligence, and sophistication. Capitalizing on the prospects for energy-efficiency enhancements and consumption upgrades, and amid formidable market competition, the enterprise harnessed the “Industrial Internet + Home Appliances” paradigm to aggregate diverse resources and forge an innovative ecosystem network. The enterprise also prioritized organizational strategy. Despite the inadequacy of extant technological prerequisites to underpin enterprise digitization, under strategic tutelage, it the enterprise has transitioned from having deficient to ample technological reservoirs, culminating in the realization of digital-servitization endeavors. This has facilitated digitization and exemplified the typical characteristics of the organizational-environmental synergistic configuration.

### 5.3. Technology-organization collaborative oriented pathway

Within Configuration 3, the core conditions encompassed the presence of a digital platform and a heightened level of financial backing, with an elevated degree of talent-system construction serving as an edge condition. Configuration 4 delineated its core conditions as extensive digital-platform utilization, robust financial support, and robust talent-system construction, with heightened product-technology iteration constituting an edge condition. These two configurations suggest that in contexts where market competition is inadequate, manufacturing enterprises can attain heightened levels of digitization by integrating product-technology iteration and talent-system construction with the utilization of digital platforms and ensuring robust financial backing. Particularly, amid uncertain levels of market competition, manufacturing enterprises, spurred by an internal impetus to forge pioneering technological competencies and competitive edges [53], accord primacy to internal organizational enhancement. Furthermore, amalgamating digital-platform advancement facilitates accessibility to a plethora of innovative resources, fostering synergistic innovation, mitigating information asymmetry, expediting the pace of digital innovation, and engendering a competitive edge. This configuration archetype corroborates the significance of product-technology iteration in propelling digital innovation within manufacturing enterprises, underscoring the collaborative impacts of organizational-management prowess and technological adeptness in fostering digital innovation.

A typical case for Configuration 3 is Sailun Group, in the rubber industry manufacturing sector. Sailun Group stands as a pioneering entity in the production of industrial control-system apparatuses within China, facilitating the intelligent advancement of industrial and affiliated domains. Amid the expansive vistas offered by the contemporary technological revolution and industrial metamorphosis propelled by big data and intelligence, the organization bolstered market-expansion endeavors through the sustenance of an all-encompassing technical innovation cadre, spanning from principal authorities to technical luminaries. With 35.9% of its workforce possessing a bachelor’s degree or higher, and leveraging a formidable assemblage of top-tier talents alongside the “Eco-rubber Cloud” Industrial Internet platform, the enterprise constructed a comprehensive scientific and technological knowledge repository. The organization optimized the harnessing of both internal and external resources to engender the requisite competencies essential for the attainment of elevated levels of digital-service innovation. Moreover, situated amid the industrial milieu of the National Rubber and Tire Engineering Technology Research Center demonstration base, the corporation has reaped the rewards of a more inclusive innovation ambiance and governmental transformation fund backing.

A paradigmatic exemplar of Configuration 4 is the Midea Group, within the white goods manufacturing sector, which stands as a global technological behemoth, spanning five business verticals: smart home, building technology, industrial technology, robotics and automation, and digital innovation. As a vanguard entity, Midea leverages its technological research and development prowess and talent reservoir. The organization has established both a talent cadre and a management ensemble steeped in a culture of technological innovation. Abiding by an autonomous innovation strategy and maintaining a steadfast commitment to incessant investment in research and development resources, the enterprise commenced the development of an Industrial Internet platform—M loT Midea, culminating at the close of 2018. Leveraging complementary strengths, it also built an industrial Internet platform characterized by openness, high flexibility, modularity, upgradeability, and autonomous control. Both Sailun Group and Midea Group have adhered to the amalgamation of dual transformations and the fusion of intelligent manufacturing, harnessing the empowering impact of digital technology on organizational paradigms.

## 6. Conclusion

### 6.1. Findings

In this study, we employed the TOE theoretical framework and synergized it with the dynamic landscape of transformation and evolution witnessed within Chinese manufacturing enterprises. Accordingly, we constructed a path model delineating the antecedent configurations underpinning the digital-servitization transformation within said enterprises. The framework elucidated the trajectory options pertinent to the digital-servitization transformation observed within manufacturing enterprises, thereby addressing the imperative for these enterprises to harmonize with their developmental milieu and select transformational stratagems and methodologies congruous with enduring expansion. The primary discoveries were as follows.

(1) Multifaceted synergy as a catalyst for transformational success: This investigation unveiled the inadequacy of individual variables in propelling the triumphant digital-servitization transformation within manufacturing enterprises. Optimal facilitation of transformation mandated the harmonious evolution of myriad factors. This underscored the imperative for manufacturing enterprises, amid the digital epoch, to pursue transformation by harmonizing with the prevalent customer-centric service-dominant paradigm [54]. As such, due regard must be accorded to synchronizing internal and external verities, choreographing resources, and engendering varied scenarios.

(2) Pluralistic transformation trajectories: The extant transformation routes for Chinese manufacturing enterprises chiefly encompassed the TOE-oriented, organization-environment collaborative-oriented, and technology-organization collaborative-oriented pathways. This illustrated the potential contribution of manifold trajectories to the digital servitization of manufacturing enterprises, epitomizing the concept of diverse paths converging at a unified destination. Given resource limitations, manufacturing enterprises should align their transformational ambitions, prioritize the cultivation of pivotal technologies or fortification of organizational governance [55], accentuate market competitiveness amid the perpetually shifting milieu, forge a dynamic rapport with clientele, construct and continuously refine customer-centric scenarios, cater to dynamically evolving customer requisites, bolster customer loyalty, and enhance the enduring efficacy of service innovation.

(3) Embracing the digital economy and shattering conventions: This study underscored the imperative for manufacturing enterprises to overcome their constraints and embrace the digital economy. A comprehensive examination of case enterprises revealed that certain entities, despite possessing weak technological prowess or encountering limited environmental support, adeptly harnessed extant resources and continually surmounted resource constraints to realize digital service-oriented progress. Furthermore, manufacturing enterprises should fully exploit the "propulsive effect," steadfastly capitalizing on their product-technology iteration capabilities, as well as perpetually innovating technological advancements and organizational governance, thus fostering the attainment of digital service-oriented organizations. Manufacturing enterprises should also proactively adapt to internal and external environmental shifts, bolster process empowerment, fortify the expansion and interconnection of the industrial chain and regional innovation ecosystem, reconfigure the enterprise’s value chain [56], and expedite the digital-servitization transformation process.

### 6.2. Contributions

This study made the following contributions.

(1) Our configurational analysis of the six key factors significantly enriched the body of theoretical research on digital servitization within manufacturing enterprises. While existing studies have predominantly focused on digital innovation or servitization in digital enterprises [57, 58], there has been a notable dearth of research specifically addressing digital servitization in the manufacturing sector. Given the manufacturing industry’s pivotal role in the national economy, a theoretical discussion on the pathways to effective digital servitization serves as a valuable guide for manufacturing enterprises. Examining the research methodology employed, current literature tends to rely heavily on case studies when investigating the influencing factors of digital servitization, with empirical research being comparatively scarce. In this study, leveraging data from 28 manufacturing enterprises and employing the TOE framework, we conducted a configurational analysis of the six factors influencing digital servitization in manufacturing. This endeavor not only augmented targeted inquiry on digital servitization within manufacturing but also elucidated the synergies among disparate factors. Our approach aligned with the recommendation by Tronvoll et al. [2] to employ QCA methods in exploring factors influencing digital servitization. Furthermore, we corroborated the influence of factors such as digital-platform construction and market competition on digital servitization, furnishing invaluable insights for future research in the realm of enterprise digital servitization.

(2) This study enriched enterprise transformation theory by diversifying the vantage point on digital servitization within manufacturing. Furthermore, we veered away from a singular viewpoint, such as digital-platform construction or organizational management, and instead moved toward a holistic perspective that contemplated the synergistic effects of technology, organization, and environment. Additionally, by leveraging synergy theory and the TOE framework, we concurrently scrutinized six factors influencing digital servitization in manufacturing. We discerned three configurational pathways capable of propelling high-level digital servitization in manufacturing, unveiling the synergistic effects and reciprocal alignment patterns among myriad conditions like technology, organization, and environment that catalyzed digital innovation in manufacturing enterprises. This all-encompassing perspective engendered a more nuanced exploration of digital servitization in manufacturing, surmounting limitations ingrained in studies exclusively fixated on individual factors and their ramifications or influence mechanisms. Furthermore, the present study broadened the application scope of the TOE framework in elucidating digital servitization in manufacturing.

### 6.3. Policy implications

The policy implications are as follows.

(1) Manufacturing enterprises should accord priority to the role of talent-system development in digital servitization. Talent-system development emerged as either a core or marginal condition in three out of the four high-level digital-servitization configurations. This underscores the profound influence of talent-system development on digital innovation within manufacturing enterprises. A highly proficient workforce facilitates the seamless integration of innovation resources and the exploration of innovation potential, thereby propelling digital servitization. Consequently, manufacturing enterprises need to acknowledge the pivotal significance of human capital as a crucial competitive advantage.

(2) The application of digital platforms could markedly contribute to the progression of digital servitization in manufacturing enterprises. In three out of the four configurations portraying high-level digital servitization in manufacturing, digital platform construction emerged as a core condition. Thus, manufacturing enterprises should proactively embrace digital platforms to lay the groundwork for digital innovation. Leveraging digital platforms could facilitate collaborative cross-organizational network engagements, streamline the innovation process, and harmonize internal and external resources to foster competitive products. Government entities should also foster institutional environments conducive to the participation of manufacturing enterprises in digital platforms, thereby bolstering their zeal for innovation.

(3) Manufacturing enterprises should grasp the importance of configurational coordination. By adopting a holistic perspective and considering practical realities, a synchronized alignment of factors such as technology, organization, and environment should exist. Pioneering optimal pathways should lead to converging outcomes. For manufacturing enterprises grappling with fierce market competition yet lacking technological prerequisites, emphasis should be placed on internal organizational restructuring to meet the exigencies of digital innovation. This necessitates the establishment of seamless internal and external connections to procure external resources. In instances where talent-system development is wanting, manufacturing enterprises should concentrate on enhancing internal technological capabilities and organizational facets. This can be accomplished through augmented research and development outlays and the attraction of top-tier talent to actualize digital servitization. Furthermore, in industries characterized by subdued vigor or situated in non-first-tier cities, manufacturing enterprises can still achieve digital servitization by leveraging a blend of technological and organizational conditions.

### 6.4. Limitations and prospects

First, while the selection of influencing factors was grounded in the TOE framework and exhibited a degree of scholarly rigor, this could lead to the omission of factors outside this framework, such as consumer demand. Subsequent research endeavors can delve into alternative theories to further explore these overlooked factors. Second, although the measurement methods utilized in this study are widely employed in academia, the continuous evolution of measurement techniques necessitates perpetual refinement. For example, while the measurement of digital servitization accentuated comprehensiveness, it fell short of a fully exhaustive array of metrics. Subsequent research endeavors can deploy more fitting measurement instruments or indicators for a more thorough examination. Finally, the study’s sample size was comparatively modest, and we solely undertook a static examination of the configurational pathways of digital servitization in manufacturing enterprises. Despite the contemporaneity of the data, subsequent research endeavors can leverage the QCA method to include larger samples and contemplate temporal factors for a more holistic investigation of digital servitization.
